# Radiation-Induced Fibrosis (RIF) in Head and Neck Squamous Cell Carcinoma (HNSCC): A Review

**DOI:** 10.3390/cells14241969

**Published:** 2025-12-11

**Authors:** Molly E. Muehlebach, Sidharth Pradeep, Xin Chen, Levi Arnold, Anna E. Arthur, Gregory N. Gan, Sufi Mary Thomas

**Affiliations:** 1 Department of Otolaryngology—Head and Neck Surgery, University of Kansas Medical Center, Kansas City, KS 66160, USA; 2 Department of Dietetics and Nutrition, University of Kansas Medical Center, Kansas City, KS 66160, USA; 3 Department of Radiation Oncology, University of Kansas Medical Center, Kansas City, KS 66160, USA

**Keywords:** radiation-induced fibrosis, head and neck squamous cell carcinoma, radiation therapy

## Abstract

Radiation-induced fibrosis (RIF) refers to the aberrant and continuous induction of myofibroblast-mediated wound healing in response to radiation therapy (RT) and occurs in up to 50% of head and neck squamous cell carcinoma (HNSCC) patients post-RT. Frontline treatment consists of an anti-inflammatory agent, pentoxifylline, in combination with an antioxidant, Vitamin E, (PENTOX) along with palliative care agents such as corticosteroids, non-steroidal anti-inflammatory agents, muscle relaxants (i.e., botulinum toxin A), or physical therapy for alleviation of symptoms such as pain, inflammation, and lymphedema. However, while efficacious in stabilization and palliation of disease, PENTOX is one of the only established agents with confirmed anti-fibrotic effects in HNSCC. Alternative therapies such as hyperbaric oxygen therapy or superoxide dismutase show efficacy in alleviating acute radiation toxicities but lack a substantial reduction in fibrotic burden. Furthermore, experimental investigations into natural antioxidants, anti-fibrotic agents approved for idiopathic pulmonary fibrosis, mesenchymal stem cell therapy, and general nutritional support, indicate anti-RIF potential, but studies in HNSCC specifically are lacking. This review aims to characterize the pathogenesis of RIF development in the HNSCC disease setting and summarize promising anti-fibrotic agents under investigation for radiation-induced toxicities.

## 1. Introduction

Head and neck cancer (HNC) is the seventh most prevalent form of cancer worldwide, accounting for up to 4.8% of cancer deaths each year [1,2]. The majority of HNC cases are squamous cell carcinoma (HNSCC) originating from the mucosal epithelium of the oral cavity and pharynx [3]. Treatment for HNSCC consists primarily of surgery followed by post-operative chemoradiation, with this regimen shown to be curative in some patients. Over the years, refined surgical and radiotherapy techniques, with the addition of immunotherapy in the metastatic setting and better diagnostic strategies, have further improved overall survival and patient outcomes in HNSCC. However, epidemiological studies are reporting an increase in global incidence of up to 30% by 2030, indicating a need for continued research efforts in this field [4,5,6]. Risk factors for disease primarily include alcohol and tobacco use and/or human papillomavirus (HPV) infection, with rising incidence of HPV in the United States and Europe suspected to account for the epidemiological rise in occurrence [1]. However, other factors, including viral infections (i.e., Epstein–Barr virus (EBV), human immunodeficiency virus (HIV), areca nut use, poor oral hygiene, and genetic predisposition, are also known to contribute to disease development and severity [1]. HNSCC is commonly associated with mutations in genes such as *TP53*, *CDKN2A*, and *PIK3CA*, but the only official diagnostic biomarker is HPV DNA p16 in the case of HPV-positive patients [7,8]. Beyond HPV, response to targeted therapies, such as epidermal growth factor (EGFR) monoclonal antibody, cetuximab, and immune checkpoint inhibitors (ICI, i.e., nivolumab and pembrolizumab), has indicated a prognostic role of EGFR and PD-L1 in disease [8,9,10]. Frontline treatment for HNSCC consists primarily of surgical intervention in conjunction with postoperative radiation and/or chemotherapy. However, while efficacious in targeting disease, radiotherapy (RT) is commonly associated with severe long-term side effects.

RT refers to the use of ionizing radiation to induce sublethal DNA damage, which, if unrepaired, leads to tumor cell apoptosis [11]. The original standard of care consisted of fixed-beam conventional RT (3D-CRT), which was used more commonly around 15–20 years ago but is no longer routine in most of the developed world. Modern standard-of-care RT for the management of HNSCC utilizes intensity modulated RT (IMRT) and is often delivered in a 360º helical fashion (VMAT), allowing clinicians to escalate RT dose to the tumor target while simultaneously sparing and/or reducing RT dose to critical organs at risk (i.e., salivary glands, constrictor muscles, spinal cord). This type of RT (IMRT-VMAT) requires the use of computed tomography and advanced computing to identify the appropriate beam arrangements and dose intensity used when designing an RT treatment plan. Numerous articles have demonstrated that IMRT-VMAT has significantly reduced xerostomia and dysphagia side-effects for HNSCC patients compared to 3D-CRT [12,13]. But despite these improvements in RT delivery and targeting, acute and chronic toxicity have remained an issue, and these toxicities are worsened with the addition of radiosensitizing chemotherapy.

Acute and long-term RT-associated toxicities are described in Table 1, with oral mucositis being the most prevalent side effect in HNSCC (occurs in up to 90% of patients undergoing RT) [14,15,16]. Other acute toxicities include changes in taste and vocal tone, local inflammation in the skin and parotid gland, dysphagia, and lymphedema. Most of these acute toxicities improve following RT. However, chronic side effects of RT, such as xerostomia, trismus, and osteoradionecrosis (ORN), are much harder to treat due to the gradual loss of parenchymal and stem cell populations, and are substantially exacerbated with combined chemotherapy. This is especially true in the case of radiation-induced fibrosis (RIF).

RIF is one of the most prevalent long-term effects of RT in HNSCC, with grade 2–3 incidence of fibrosis based on the Radiation Therapy Oncology Group (RTOG) grading scale occurring in up to 30–50% of HNSCC patients [17]. RIF can occur in the muscles surrounding the neck (i.e., sternocleidomastoid, platysma), jaw (i.e., mastication muscles), and base of the tongue, contributing to trismus and impacting daily activities such as speech and swallowing [16]. RIF also commonly occurs in the skin and subcutaneous tissues, in which hardening of the tissue leads to stiffness and cosmetic changes [18]. Symptoms consist of induration, telangiectasia, pigmentary changes, and the formation of ulcers on the superficial face and neck tissue, further contributing to reduced quality of life in these patients [18]. Additionally, fibrosis can occur in the lymphatic system, leading to lymphedema and swelling in the face and neck, or affect the salivary glands, exacerbating xerostomia [16]. In some cases, the pharynx and esophagus are also affected, increasing the risk for pharyngeal stenosis and the occurrence of mucositis [16].

Established therapies for RIF in HNSCC consist primarily of an anti-inflammatory agent, pentoxifylline (PTX), in combination with an antioxidant, Vitamin E (PENTOX). Specifically, PENTOX has been found to reduce the fibrotic area of RIF lesions; however, complete abrogation of fibrosis is yet to be seen. Investigation into therapies such as hyperbaric oxygen (HBO) therapy or superoxide dismutase (SOD) has shown benefit in alleviating ORN, xerostomia, and dysphagia, but they do not exhibit substantial anti-fibrotic effects. The purpose of this review is to characterize the pathophysiology of RIF in HNSCC and further summarize investigative therapies with translational potential in this cohort of oncology patients. Of note, this is a narrative review due to the lack of substantial clinical and preclinical studies evaluating anti-fibrotic therapies for RIF in HNSCC-specific settings. This review discusses the well-established molecular mechanisms involved in RIF pathogenesis, summarizes the minimal clinical evidence of RIF ameliorating agents in HNSCC patients, and, furthermore, reviews the available literature involving non-HNSCC-specific RIF model systems to expand upon potential anti-fibrotic therapies for future investigation in HNSCC RIF.

## 2. Mechanistic Insights

Unlike other cancers, the HNSCC tumor stromal microenvironment is especially rich in tumor fibroblasts, also known as cancer-associated fibroblasts (CAFs) [19]. While multiple types of CAFs have been identified, myofibroblasts appear to be the predominant fibroblast cell type residing in the HNSCC tumor microenvironment (TME) [20]. Specifically, irradiated CAFs-myofibroblasts have been shown to undergo metabolic and epigenetic reprofiling to promote extracellular matrix (ECM) production and secretion of various pro-tumorigenic soluble factors that mediate tumor growth, suppress the immune response, and facilitate tumor metastases [20]. Neutrophils are one of the first immune cells to respond to RT-induced signals, arriving at the site of radiation damage to release pro-fibrotic signals such as interleukin 1β (IL-1β) and interleukin 6 (IL-6) [21,22]. Monocytes differentiated into M2-like macrophages are next to localize where they produce growth factors such as platelet-derived growth factor (PDGF), connective tissue growth factor (CTGF), and TGFβ1 to induce fibrogenesis [22,23]. Importantly, TGFβ is considered a master regulator of fibrosis due to its regulation of fibroblast proliferation, differentiation, and function [24]. Specifically, TGFβ is reported to signal for the proliferation of postmitotic fibrocytes from progenitors and induce local fibroblast-to-myofibroblast activation [25]. Furthermore, TGFβ regulates ECM deposition by inciting both ECM protein production and secretion (i.e., collagen, fibronectin, proteoglycans). TGFβ also reduces ECM turnover by promoting activation of tissue inhibitors of metalloproteinases (TIMP), and concurrent downregulation of matrix metalloproteinases (MMP) [26,27]. In aggregate, these signaling molecules contribute to the chronic disruption of balanced ECM synthesis and degradation, resulting in tissue stiffness, poor vascularity, necrosis, and eventual organ failure due to RIF [28,29].

**Figure 1:**
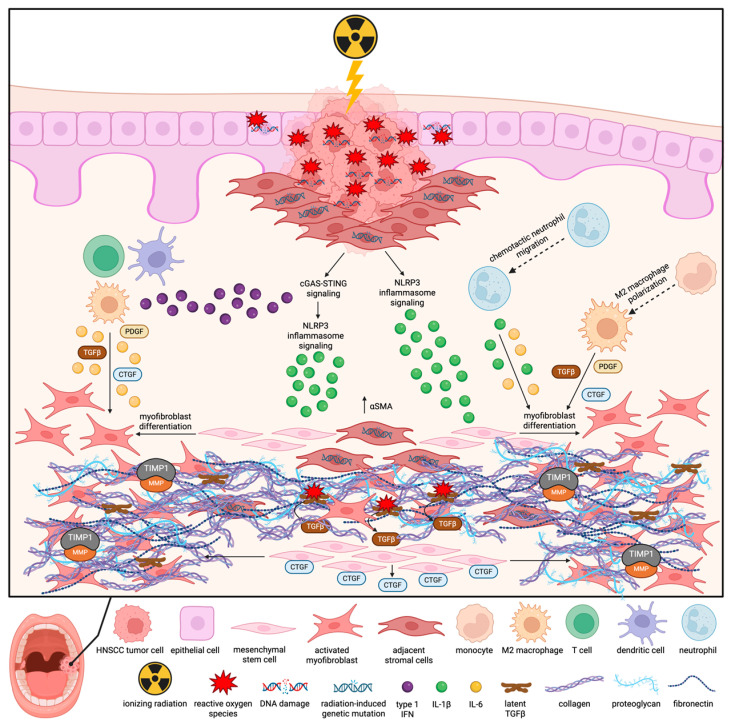
Pathogenesis of RIF in HNSCC. Ionizing radiation produces reactive oxygen species (ROS) and DNA damage in HNSCC tumor cells and adjacent healthy tissue (i.e., epithelial, stromal cells). Accumulation of cytosolic DNA damage activates cGAS-STING signaling, inducing the type I interferon (IFN) response and NLRP3 inflammasome activation. IFN-mediated recruitment of innate and adaptive immune cells mediates secretion of pro-fibrotic cytokine, IL-6, and fibroblast growth factors such as platelet-derived growth factor (PDGF), connective tissue growth factor (CTGF), and transforming growth factor β (TGFβ). ROS signaling induces NLRP3 inflammasome activation and secretion of IL-1β. Infiltrated immune cells, such as neutrophils and M2 macrophages, promote fibroblast recruitment and activation via secretion of IL-6 and growth factors like PDGF, CTGF, and TGFβ. Radiation exposure to adjacent stromal cells causes genetic mutation, increasing expression of myofibroblast marker, α-smooth muscle actin (αSMA), and promoting extracellular matrix (ECM) production. ROS also promotes fibrosis through activation of latent TGFβ in the ECM. This creates a positive feedback loop by which TGFβ positively regulates CTGF production to continuously promote fibroblast proliferation and activation. Furthermore, TGFβ promotes activation of tissue inhibitors of metalloproteinases (TIMP) to disrupt matrix metalloproteinase (MMP) activity, leading to excess ECM and fibrosis. Created with Biorender.com.

While RIF-mediating pathways specific to HNSCC pathology have yet to be fully characterized, malignant pathways mediating RIF, in general, are becoming clearer [22,30]. A major mediator of RIF is oxidative stress due to the induction of pro-inflammatory and fibrinogenic signals [31,32,33]. As ionizing radiation interacts with water molecules in the cell, hydroxyl radicals and superoxide ions are created while also destroying cellular scavengers of ROS, such as glutathione peroxidase and SOD [34]. Saturated intracellular levels of ROS can then activate NOD-like receptor protein 3 (NLRP3) inflammasome pro-inflammatory signaling and production of pro-fibrotic factor IL-1β [32,33]. Furthermore, ROS can oxidize the cysteine residues of latency-associated peptides (LAP), allowing for latent TGFβ in the ECM to become active [35]. This mediates a positive TGFβ feedback loop through Rho/Rho-associated protein kinase (RHO/ROCK) induction of CTGF for continued myofibroblast activation [34,36]. In addition, ROS has been found to epigenetically modify fibroblast gene expression such that α-smooth muscle actin (*αSMA*) expression is upregulated to induce myofibroblast transformation [34,37]. Therefore, in the case of RT, where patients are repeatedly exposed to RT injury, chronic activation of NLRP3 signaling and TGFβ-mediated positive regulation of fibrogenesis, constitutively favors fibroblast activation and ECM secretion, contributing to RIF development (Figure 1) [34].

Importantly, ionizing radiation produces not only ROS but also DNA damage such as single-strand breaks (SSB), double-strand breaks (DSB), and nucleotide base damage. Evidence has shown that the degree of DNA damage varies based on the type of radiation and dosage [38]. Specifically, the degree of linear energy transfer (LET), referring to the rate of energy that a charged particle transfers to the material it passes through per unit of distance, directly impacts DNA damage [38]. High doses of low-LET photon therapy or high-LET proton therapy have been shown to cause clustered or complex DNA damage (CDD), consisting of two or more types of DNA damage occurring in close proximity on the same DNA strand [39]. CDD is substantially more difficult for the cell to repair and is, therefore, commonly mis-repaired or left unrepaired as residual damage [39]. As cells with CDD attempt to replicate, damaged DNA fragments accumulate in the cytosol or cluster with nuclear envelope proteins to form micronuclei [40]. DNA sensors such as cyclic GMP-AMP synthase (cGAS) and absent in melanoma 2 (AIM2) can then detect these fragments and trigger stimulator of interferon genes (STING) dependent type I interferon (IFN) and nuclear factor-κB (NF-κB) inflammatory signaling, activating the innate and adaptive immune response [40,41]. While this is beneficial in regard to anti-tumor activity, prolonged activation promotes M2 macrophage polarization and continuous production of inflammatory molecules such as IL-6 and NLRP3 inflammasome activation, linking RT insult to pro-fibrogenic remodeling (Figure 1) [42].

Furthermore, RT-induced ROS and DNA damage have been shown to activate cellular senescence, consisting of cell cycle arrest via p53-mediated activation of p21, and p16-mediated phosphorylation of retinoblastoma protein (Rb) [43]. The purpose of cell cycle arrest is to downregulate functional cellular processes to allow for restoration of DNA damage and induction of antioxidant enzymes such as SOD and glutathione peroxidase; however, persistent senescence due to repeated RT exposure can favor a senescent-associated secretory phenotype (SASP) [43]. SASP-transformed cells favor pro-inflammatory signaling cascades through the secretion of cytokines such as IL-6, IL-8, IL-1β, and IFN, promoting myofibroblast differentiation and recruitment of TGFβ-secreting macrophages [43,44]. Therefore, senescence further contributes to fibrotic disease and severity.

## 3. Methods for Diagnosing and Studying RIF in HNSCC

Considering anti-fibrotic therapies to be most efficacious in the acute setting, stratification and classification of fibrotic lesions could provide prognostic and treatment-specific insight for patients with RIF. Grading systems for RIF stratification consist of qualitative, subjective assessments such as the Common Terminology Criteria for Adverse Events (CTCAE) and RTOG-based scales, which rely on visual inspection and palpation examination to determine RT toxicities such as RIF [45]. Alternatively, the Late Effects Normal Tissue Task Force Subjective, Objective, Management, and Analytic (LENT-SOMA) score provides a more specific and detailed criterion for evaluating fibrotic symptoms but is most accurate for superficial RIF [46]. While noninvasive and simple, these assessments lack substantial objective measures and are not specific to fibrotic disease. Biopsy and histological evaluation offer an objective measure for RIF, but the anatomical location of RIF in HNSCC makes biopsy a highly invasive and painful option. As a result, quantitative conventional imaging techniques such as MRI, PET/CT, and ultrasound are commonly used for the identification of tissue thickening or changes in local vasculature, but these modalities lack precise information regarding fibrosis (i.e., early or late stage, active or stable). Newly developed techniques such as ultrasound shear wave elastography (SWE) have been shown to more accurately measure RIF in HNC patients and confirmed elevated tissue stiffness within the sternocleidomastoid muscle post-RT [47]. SWE quantifies tissue elasticity in response to an applied acoustic force, specifically measuring the tendency of tissue to resist deformation or return to its original shape after force has been applied [47]. Similarly, MRI elastography, in which external equipment is used to generate shear waves that are MRI-recorded for change in elasticity, is also an option for RIF diagnosis in HNSCC. However, while measurement of tissue elasticity is a great method for quantifying fibrotic degree, ultimately, correlation of these measures with other indicators of RIF, such as dysphagia or pain scores, would provide the best overall quantification of disease.

Non-invasive molecular probes have also been in development to target different fibrosis-specific or fibrosis-associated processes to indicate the fibrotic stage [48]. Success has been seen with probes targeting ECM proteins such as type I collagen and α_5_β_6_ integrin in patients with idiopathic pulmonary fibrosis, but the majority have remained preclinical with evaluation in mouse models of myocardial infarction, pulmonary fibrosis, liver fibrosis, and atherosclerosis [48]. EP-3533 is a peptide-based gadolinium probe specific to type I collagen, which has shown promise. Analysis in models of liver and pulmonary fibrosis has shown accurate detection of collagen deposition, which correlates with biochemical levels of hydroxyproline [49,50]. But while the use of molecular imaging probes for fibrosis stratification seems to be an accurate measure of fibrotic status, pharmacokinetic optimization of these probes in humans is still needed.

Apart from diagnostic development, model systems for studying RIF and investigative therapies are available, but research efforts have primarily been limited to studies in the pulmonary setting. In vitro modeling of RIF primarily consists of fibroblast cells exposed to ionizing radiation and/or TGFβ induction of collagen production to upregulate fibrotic genes (i.e., *mTORC2, CDKN2C*, and *ITGα1β1*) [51,52,53]. Downstream readouts for these models commonly include real-time quantitative polymerase chain reaction (RT-qPCR) and Western blotting for key fibrotic markers (TGFβ, CTGF, and αSMA), and the use of staining procedures such as Sirius red for changes in collagen production [51]. However, while these models allow for identification of key signaling proteins and soluble factors mediating RIF development, and allow for mechanistic elucidation of potential anti-fibrotic compounds, they fail to consider effects of the tumor microenvironment (TME) (i.e., infiltrating immune cells) and lack HNSCC cells, which are the primary target of RT. Considering it is the tumor response to ionizing RT that mediates pro-inflammatory and fibrotic signaling cascades, the use of co-culture models or 3D model systems represents a more translational option.

Newly developed 3D organoid models of HNSCC could be beneficial for studying RIF development by recapitulating the physiochemical and cell–cell interactions found in vivo [54,55]. This provides a benefit in studying changes to ECM composition, considering that turnover is directly influenced by local cell interactions and spatial organization. Furthermore, the use of patient tissue for these models allows for replication of the genetic, histological, and functional aspects of disease, which contribute to RT response and toxicities. However, these models are still limited in that spatial distribution may not directly replicate that of the tumor in humans and lack integration of physiological cues from outside organ systems, which may contribute to RIF. While 3D organoid models have been used for fine-tuning RT dose in HNSCC disease, application for developing RIF therapies has yet to be seen [54].

HNSCC model systems in vivo, which consist of tumor transplantation (allograft and xenograft), chemically induced carcinogenesis, and genetically modified animal models, have also been developed for studying RIF [53,55,56]. Patient-derived xenograft (PDX) models, which follow the transplantation of human tumor tissue to immunocompromised mice, are widely used for investigating novel HNSCC agents [53,55]. However, PDX models lack immune cells, which mediate activation and secretion of pro-inflammatory/pro-fibrotic signals that mediate myofibroblast differentiation and ECM secretion. This ultimately limits the use of these models when evaluating anti-fibrotic HNSCC therapies. Alternatively, syngeneic models in which mouse HNSCC cells are engrafted in the oral cavity (i.e., floor of the mouth) or exposure of mice to carcinogens such as 4-nitroquinoline 1-oxide (4-NQO) via drinking water to induce oral tumor formation, offer viable immunocompetent models in which mice can be treated similarly to HNSCC patients in the clinic with surgical tumor resection and post-operative RT [55]. Furthermore, genetically engineered mouse models (GEMM), in which various HNSCC-associated mutations are transduced, resulting in spontaneous tumor formation, are efficient in that they recapitulate tumor initiation and progression in an immunocompetent environment [56]. Evaluation of anti-fibrotic agents in these settings could be beneficial for evaluating alterations to innate and adaptive immune signaling and quantifying changes in fibrotic burden; however, limitations include a lack of representation of human HNSCC and variations between human vs. murine biology. For example, biological response to RT has been shown to vary across mouse strains, with certain strains exhibiting resistance to fibrosis development [57]. Furthermore, the choice of RT dose and delivery technique will significantly influence experimental outcomes, indicating a need for optimization and confirmation of appropriate biological response before the use of these models for therapeutic development.

## 4. Conventional Therapies for RIF in HNSCC

As previously mentioned, frontline treatment for RIF in HNSCC consists of PENTOX therapy combining an anti-inflammatory agent, PTX, with Vitamin E. While the anti-inflammatory mechanism of these agents is known to disrupt production of TGFβ, suggesting direct anti-fibrotic potential, complete abrogation of RIF in HNSCC patients is yet to be seen with this therapy [58,59,60]. Botulinum toxin A (BTA), cyclobenzaprine (Flexeril), anxiolytics such as lorazepam, and physical therapy (PT) are also common interventions post-RT due to their muscle relaxant and pain-relieving benefits [14,61,62]. However, while these treatments have been investigated, as to alleviation of symptoms such as dystonia, trismus, and neck pain, overall benefit depends on the timeline and frequency of intervention (during or post-RT) [63,64,65,66,67,68].

PTX is a non-selective phosphodiesterase inhibitor that increases cellular levels of cyclic adenosine monophosphate (cAMP) to modulate target signaling pathways [69]. Studies on dermal fibroblasts in vitro revealed that PTX inhibits IL-1-induced fibroblast proliferation and TNFα-induced fibroblast activation [70,71]. Furthermore, PTX upregulated collagenase activity and markedly reduced the amount of collagen, glycosaminoglycans, and fibronectins [70,72]. Clinically, PTX is used in combination with an antioxidant, Vitamin E (α-tocopherol), which acts as a ROS scavenger to protect against oxidative stress. Studies evaluating combined PTX and Vitamin E (PENTOX) in HNSCC are limited, with most studies focusing on efficacy in breast cancer (BC). However, an early study in BC and HNC patients suffering from RIF found reduced total fibrotic area (up to 50% after six months) and improved SOMA injury scores with amelioration of edema, local inflammation, and restricted movement [73]. Furthermore, PENTOX has been found to improve acute RT symptoms such as dysphagia, dysgeusia, and oral mucositis, suggesting a broader protective benefit against RT exposure [74]. A more recent study by Harpso et al. evaluated PENTOX treatment in BC and HNC patients and reported a 75% response rate in BC patients compared to 23% in HNSCC [75]. Of note, BC patients in this study received a lower dose of RT compared to HNC patients, but this data emphasizes the importance of studying RIF therapies in HNSCC-specific settings due to variation in tissue-specific RT effects and treatment response [75]. Overall, these studies highlight variation in clinical outcomes across cancer types, indicating tissue, previous RT exposure, time to intervention, and location of fibrosis (cutaneous/superficial vs. subcutaneous) to impact PENTOX efficacy (Table 2).

The pro-vascular effects of PTX alone and in combination with Vitamin E have been found to promote mucosal healing of necrotic tissue, presenting a special benefit in HNSCC due to the development of ORN [76,77,78]. ORN arises from necrosis of mucosal tissues, hypoxia due to loss of vasculature, and disruption of normal bone remodeling, all resulting from RT exposure [79]. RIF contributes to ORN morbidity by physically impeding infiltration of new blood vessels and promoting poor mandible integrity [79,80]. Therefore, suggesting benefits from PTX are due to both anti-fibrotic and pro-vascular mechanisms, attenuation of ORN, and overall improvement of long-term RT effects [76,77,78,81]. PENTOX benefits to ORN have been most notable when used in combination with an FDA-approved bisphosphonate, clodronate [78,82,83,84,85,86,87,88,89]. Clodronate is reported to stimulate osteoblast bone formation and inhibit macrophage (primary source of TGFβ) infiltration, reducing fibroblast proliferation [90,91]. Therefore, adding clodronate to PENTOX regimens may promote anti-fibrotic effects [78,92]. Furthermore, prophylactic use of PTX or PENTOX has been shown to prevent the incidence of soft tissue necrosis, while a prophylactic Vitamin E oral rinse reduced the risk of symptomatic mucositis by 36% [93,94,95]. Even prophylactic BTA has been shown to protect against salivary gland damage, suggesting benefit in ORN [96].

RT-induced hypoxia is a benefit in that it disrupts nutrient flow to the tumor; however, disruption of blood flow to surrounding healthy tissues contributes to ORN, as previously mentioned. HBO therapy involves intermittent inhalation of 100% oxygen above normal atmospheric pressure (i.e., >1 atm). This enhances available oxygen levels to damaged tissue, expected to facilitate angiogenesis and wound healing, but importantly, does not promote tumor growth [97,98]. HBO treatment in HNSCC patients post-RT has been shown to improve long-term side effects of RT such as ORN, xerostomia, dysphagia, and overall quality of life. However, direct anti-fibrotic effects have not been reported [99,100,101,102]. Meta-analysis of HBO use during RT showed improvement in local tumor control, mortality, and tumor recurrence in HNC, which has supported its continued investigation to help with mitigating late HNSCC RT complications (xerostomia—NCT02450305, ORN—NCT06055257) [103].

Abrogation of RIF has been seen with antioxidant treatment like Vitamin E, but also with antioxidant enzymes such as metalloenzyme SOD [104]. SOD is responsible for disposing of superoxide radicals in the cell; therefore, SOD formulations have been developed for decomposing RT-induced ROS and abrogating activation of fibrosis [105]. In a clinical trial (NCT01771991), sodermix, a topical formulation of SOD, was evaluated in HNSCC patients with RIF, and while 46% of treated patients showed improvements in fibrotic grade, improvement in the placebo arm was nearly equivalent (43%), suggesting benefits were due to daily massage in the fibrotic area (Table 2) [106]. However, while sodermix may present an anti-fibrotic option for superficial RIF, efficacy with RIF in deeper tissues would require further investigation. Interestingly, all participants reported reduced pain regardless of treatment, highlighting the importance of physical therapy in improving quality of life in these patients [106]. Other antioxidant formulations have been shown to reduce radiation dermatitis and oral mucositis in HNSCC patients, but effects on fibrosis were not evaluated [107,108].

Statins are widely prescribed cholesterol-lowering agents with inhibitory effects on RHO/ROCK signaling, reported to mitigate CTGF signaling in RIF [109,110]. Evidence of RIF attenuating effects in preclinical models led to evaluation in a phase II clinical trial (NCT01268202) of HNSCC patients with grade 2 or higher fibrosis according to CTCAE scoring (Table 2). Pravastatin treatment was found to induce structural improvement of the skin in 14 of 19 patients, with 8 of the 14 indicating reduced collagen infiltration and RIF thickness [111]. Therefore, future studies plan to evaluate pravastatin treatment in a phase III trial.

**Table 2:** Clinical trials evaluating anti-fibrotic therapies in previously RT treated HNC patients.

Treatment Regimen	Patient Cohort	RIF Presentation	Outcome	Reference
PENTOX (PTX 800 mg/d; Vit E 1000 U/d) administered orally for at least 6 months	Breast, and HNSCC patients (N = 43)	Symptomatic RIF involving the skin and underlying tissues occurring within 1 year of RT (ranging from 45 to 75 Gy)	53% mean reduction in fibrosis surface area after 6 months; mean linear dimensions diminished from 6.5 to 4.5 cm; SOMA injury score improved from 13.2 to 6.9 after 12 months	Phase 2 trial [73]
PENTOX (PTX 400 mg; Vit E 290–350 mg) administered orally twice daily	Breast and HNSCC patients (N = 54)	Severe and symptomatic RIF (Breast cancer patients N = 24; mean RT dose 45.7 Gy; mean duration of 31 months prior to intervention) (Head and neck cancer patients N = 30; mean RT dose 67.8 Gy; mean duration of 42 months prior to intervention)	75% reported subjective improvement in RIF as reported by LENT-SOMA score in breast cancer patients after 14 months; 23% reported subjective improvement in head and neck patients after 12 months	Retrospective study [75]
Sodermix cream (280 U/g superoxide dismutase) once daily on the fibrotic area for 3 months	HNSCC patients (N = 68)	86% of patients received RT more than 6 months before treatment initiation, with the majority having grade 1–2 fibrosis (according to CTCAE scoring)	46.6% improvement in study arm vs. 43.3% in placebo control	Randomized, prospective, blinded study [106]
Pravastatin (40 mg/d) for 12 months	HNSCC patients (N = 40)	Grade 2 or greater cutaneous and subcutaneous neck fibrosis (according to CTCAE scoring)	Ultrasonography confirmed a reduction in RIF thickness by >30% in 35.7% of patients after 12 months; the CTCAE score was improved in 50% of patients	Phase 2 trial [111]

## 5. Experimental Therapies for RIF in HNSCC

Due to the lack of substantial fibrosis attenuating effects with conventional therapies, more targeted experimental therapies have been under investigation. Unfortunately, evaluation of these agents has been limited to RT-induced pulmonary or skin fibrosis models, limiting translational efficacy in HNSCC RIF. This section aims to review the anti-fibrotic mechanisms of these experimental therapies and expand upon the potential use of these agents in HNSCC patients, with major findings summarized in Table 3.

### 5.1. Anti-Fibrotic Agents Approved for Idiopathic Pulmonary Fibrosis

Various FDA-approved therapies have undergone off-label investigation to determine efficacy in treating RIF. Agents such as pirfenidone and nintedanib, approved for treatment of idiopathic pulmonary fibrosis, have been investigated in the realm of RIF due to modulation of fibrotic signaling pathways. Pirfenidone is known to exert anti-inflammatory, antioxidant, and anti-fibrotic effects specifically through modulation of TGFβ1 [112]. Evaluation in cancer patients with RIF reportedly improved range of motion; however, direct measures of fibrosis were not evaluated [113]. Pirfenidone is still being investigated for use in HNSCC, specifically for the purpose of resensitizing cancer cells to combat CAF-mediated radioresistance (NCT06142318). Nintedanib is a multi-receptor tyrosine kinase inhibitor (RTKi), known to disrupt fibroblast activation and found to reduce RT-induced pulmonary fibrosis [114,115]. Unfortunately, application in HNSCC patients may be limited, as a case report of a patient with oropharyngeal carcinoma experienced severely delayed mucosal healing post-RT [116]. Indicating potential increased risk for ORN.

Imatinib is an RTKi investigated in HNSCC for its anti-tumoral effects (NCT00485485), which has recently been approved for study to restore cetuximab sensitivity (NCT05816785) [117,118]. Imatinib has been found to disrupt c-Abelson (c-Abl) kinase activation and PDGF signaling in fibroblasts post-TGFβ, suggesting application in HNSCC RIF [119,120]. Evaluation in pulmonary and skin RIF models has indicated attenuation of fibrosis beyond the acute toxicity phase, confirming potential application in established fibrosis, such as long-term RT patients [121,122].

### 5.2. Neutralizing Antibodies for Targeting Pro-Fibrotic Factors

As a master regulator of fibrotic disease, TGFβ-neutralizing antibodies have been developed for application in RIF. Fresolimumab is a monoclonal antibody capable of neutralizing all mammalian forms of TGFβ. Evaluation in patients with early, diffuse cutaneous systemic sclerosis demonstrated fresolimumab to decrease TGFβ-regulated biomarkers, such as NOX4, CTGF, and COL10A1 [123]. While this confirms anti-fibrotic efficacy, whether these effects are replicated in the setting of RT-mediated fibrosis requires confirmation. Furthermore, the CTGF-targeting monoclonal antibody, pamrevlumab, has been developed and investigated in models of RT-induced pulmonary fibrosis [124]. Data indicated that pemrevlumab significantly decreased lung remodeling, improving median survival in these mice, with efficacy being greater than that seen with nintedanib or pirfenidone alone [124].

### 5.3. Natural Antioxidants

Natural polyphenols such as curcumin and rosmarinic acid exert antioxidant mechanisms, showing benefits in mitigating RIF. Curcumin was found to protect against RT-induced oxidative skin injury in mice [125]; however, evaluation in treating RT-induced dermatitis in BC patients yielded no significant benefit [126]. A clinical trial (NCT05982197) evaluating curcumin oral gel in reducing RT-induced oral mucositis has been completed, with results pending. The study plans to evaluate changes in EGF and IL-8 production, which should help inform translational potential in ameliorating RIF. Rosmarinic acid is a natural polyphenol shown to reduce parotid gland tissue fibrosis in rats post-RT [127]. Importantly, rosmarinic acid was found to reverse mRNA overexpression of collagen in parotid glands, which was not seen with the clinically used agent amifostine [127]. Amifostine is one of a few approved radioprotective agents for the treatment of post-RT toxicities in HNSCC patients due to amelioration of xerostomia [128,129,130]. However, amifostine is rarely used clinically due to its high cost and common adverse reactions [131]. Evidence of fibrotic benefit with rosmarinic acid, compared to the clinically approved amifostine, supports the continued investigation of rosmarinic acid in RIF HNSCC.

### 5.4. Mesenchymal Stem Cell (MSC) Therapy

MSC therapy requires the isolation of multipotent, self-renewing stromal cells, which are then reintroduced to promote tissue healing and regeneration. Adipose-derived MSCs (Ad-MSCs) have specifically been shown to mitigate RIF through inhibition of Wnt/β-catenin signaling during fibroblast activation [132]. MSC therapy has been efficacious in treating RT-induced pulmonary fibrosis in vivo, with early phase I studies (NCT02277145) in humans indicating safety and anti-fibrotic effects [133]. Studies by Blitzer et al. confirmed the expansion capacity of bone marrow-derived MSCs from RT-treated HNC patients and reported that these cells exhibited an immunosuppressive phenotype following interferon stimulation [134]. Furthermore, injection of these MSCs into irradiated salivary glands of mice improved salivary production and reduced fibrotic burden [134]. This supported translation to clinical trials, which have now focused on MSC injection into the salivary and parotid glands of HNC patients with xerostomia post-RT (NCT02513238, NCT03876197, NCT06012604). A recently completed phase I study (NCT02513238) confirmed the safety of autologous Ad-MSC injection, which is significant due to the oncogenic risk that comes with MSC multipotency [135,136]. As studies progress, if continued efficacy is seen in mitigating xerostomia, studies should consider quantifying direct effects on fibrosis for broader clinical application. Genetic transformation of MSCs to overexpress proteins that combat RIF, such as SOD3, has been shown to more effectively attenuate pulmonary RIF opposed to unmodified MSCs, suggesting another area of investigation for this therapy [137].

### 5.5. Modulation of the DNA Damage Response

RT quality, such as the degree of LET, directly impacts the extent of DNA damage and, therefore, the repair response. High-LET, such as proton therapy, has a higher relative biological effectiveness (RBE) compared to low-LET, which is attributed to the more frequent occurrence of irreparable CDD. Low-LET RT-induced DSBs are primarily resolved through high-fidelity and low-error non-homologous end joining (NHEJ) and homologous recombination (HR) DDR pathways; however, HR is suppressed with increasing doses of low-LET RT [138]. Alternatively, canonical NHEJ is regarded as insufficient for resolution of DSBs from high-LET RT; therefore, the cell relies on alternative end-joining (alt-EJ) mediated by poly (ADP-ribose) polymerase 1 (PARP1) and single-strand annealing (SSA) for DNA repair [138]. However, these processes are more error-prone, increasing the risk of structural chromosomal abnormalities. In efforts to improve the RBE of RT, the use of DDR pathway modulators has been investigated in combination with RT and has shown a potential for improving anti-tumor response.

PARP inhibitors have been investigated for their ability to radiosensitize HNSCC cells to RT, with preclinical studies showing efficacy in enhancing tumor cell apoptosis [139,140]. However, implications on fibroblasts and RIF have yet to be investigated. While increased DNA damage may promote HNSCC apoptosis, this could suggest substantial activation of cGAS-STING inflammatory signaling, which, while beneficial for the anti-tumor response, could contribute to RIF development. A clinical trial is currently underway to evaluate the PARP inhibitor, Olaparib, in RT for HNSCC (NCT02229656). Quantification of RT toxicities like RIF in this study should help clarify whether fibrosis risk is elevated or reduced with combined PARP and RT.

Inhibition of cGAS-STING signaling has been shown to attenuate pulmonary RIF in the preclinical setting due to reduced collagen deposition and *αSMA* expression [141]. However, alternatively, STING agonists have been shown to enhance anti-tumor efficacy in combination with RT [142]. Importantly, STING signaling has been associated with SASP transformation after exposure to RT [143,144]. This is primarily thought to occur via ataxia telangiectasia mutated (ATM)-mediated phosphorylation of p53, upregulating p21 and p16 to induce cell cycle arrest in response to DNA repair [144]. Not only are senescent cells known to contribute to genotoxic resistance and cancer recurrence, but this phenotype is also associated with the secretion of factors such as IL-6, CCL5, CXCL12, CCL2, and IL-8, creating an immunosuppressive environment and promoting tissue fibrosis [145]. In conclusion, while cGAS-STING signaling may offer a therapeutic target for RIF, clarity on how the quality of RT, dosage, and temporal activation of the immune response vs. senescence impacts fibrosis development is needed.

Importantly, DNA exonucleases such as three-prime repair exonuclease 1 (TREX1) are also activated in a dose and time-dependent manner post-RT exposure to degrade cytosolic DNA, alternatively stimulating an immunosuppressive landscape and reducing the therapeutic efficacy of RT [146]. TREX1 is considered an inhibitor of cGAS-STING in that TREX1’s removal of cytosolic DNA prevents activation of this signaling cascade. TREX1 activation has been reported as a key regulator of RT-induced immunogenicity, suggesting that inhibition of TREX1 could potentially promote RT anti-tumor effects [146]. This would suggest a potential therapeutic benefit with TREX1 loss or inhibition; however, knockout of TREX1 has been shown to accelerate cGAS-STING-induced cellular senescence, indicating that the loss of this protein could potentially promote senescent-mediated pro-fibrotic processes [143,144].

**Table 3:** Summary of experimental therapies demonstrating anti-fibrotic effects in non-HNSCC-specific preclinical models of RIF.

Treatment	Anti-Fibrotic Mechanism	Anti-Fibrotic Effects in Non-HNSCC RIF Models
Pirfenidone	Inhibition of TGFβ-mediated fibroblast proliferation, myofibroblast differentiation, collagen synthesis, fibronectin production, and ECM deposition [112]	Reduced collagen deposition and fibrosis in mouse models of pulmonary RIF [147,148]
Nintedanib	Inhibits TGFβ signaling, downregulating ECM production and induction of αSMA [114]	Reduced collagen deposition in a mouse model of pulmonary RIF [115]
Imatinib	Inhibits c-Abl kinase activation of fibroblasts post-TGF*β* induction [119]	Markedly attenuated development of pulmonary RIF in mice [120]
Fresolimumab	Inhibits TGFβ-mediated pro-fibrotic effects	Reduced pulmonary RIF development in mice [149]
Pamrevlumab	Inhibits CTGF-mediated pro-fibrotic effects	Reduced lung remodeling in mice at 24 weeks post-RT [124]
Curcumin	Increases antioxidant enzymes such as superoxide dismutase (SOD), catalase (CAT), and glutathione peroxidase (GPx), ameliorating ROS-mediated fibrotic effects [125]. Shown to attenuate pro-inflammatory molecules such as NF-κB, COX2, IL-1, IL-6, IL-8, and TNFα [150]	Administration before and after RT protected against skin damage and pulmonary RIF in rats [125,150]
Rosmarinic acid	Attenuates ROS via indirect activation of the antioxidant response via PGC1-a/NOX4 and inhibition of inflammatory factors TNFα, IL-6, and IL-2, reducing collagen expression [127]	Administration protected against pulmonary RIF in rats [151]
Amifostine	Ameliorates ROS via activation of antioxidant enzymes such as SOD [152]. Reduces TGFβ-mediated signaling and pro-inflammatory cytokines such as TNFα, IL-6, and IL-1β [153,154]	Protects against pulmonary RIF and vocal fold and subglottic gland RIF in rats [152,153,154]
Mesenchymal stem cell therapy	Inhibited TGFβ activation of Wnt/β -catenin signaling, suppressing expression of *αSMA*, fibroblast proliferation, and collagen expression [132,137]	Attenuated RT-induced lung injury and fibrosis in mice [132,137]
STING inhibition	Inhibits PERK/eIF2α signaling mediating myofibroblast differentiation [141]	Knockdown of STING reduced collagen deposition and *αSMA* expression in a mouse model of pulmonary RIF [141]

## 6. Alternative Options for RIF Reduction in HNSCC

### 6.1. Alternative RT Techniques

Considering anti-fibrotic therapies for RIF in HNSCC are lacking, optimizing RT regarding the type of RT, means of delivery, and minimization of normal tissue toxicity is essential. The likelihood of RT toxicity is directly related to dose, irradiated body proportion, and tissue volume, with dosage being the most critical factor for fibrosis development [11,155]. Image-guided RT (IGRT) and image-guided adaptive RT (IGART), which allow for real-time adjustment of RT delivery according to changes in tumor volume, have further minimized RT toxicities without affecting tumor dosage [156,157]. Stereotactic body RT (SBRT) varies from conventional RT and IMRT in that it delivers a non-homogeneous dose within the target site at a higher dose per fraction while greatly reducing high dose RT to surrounding normal adjacent tissue. Use of SBRT (40 Gy/5 fractions) in locally recurrent HNSCC was associated with an incidence of late grade 3 or greater toxicities of only around 15%, with higher severity due to the re-irradiation of these tissues [158]. Importantly, SBRT has been found to have an immunomodulatory effect by promoting T-cell anti-tumor response [159,160]. To exploit this, SBRT has been under investigation in use with other ICIs, but measured outcomes such as PFS and OS did not improve with SBRT [161].

Proton therapy is another modality of RT that emits charged proton nuclei compared to photon therapy, which emits massless, uncharged photons. There is a dosimetric advantage with proton therapy because it imparts no exit dose, unlike photon-based RT, meaning the dose stops at the tumor edge, limiting effects in surrounding healthy tissue. As a result, a significant reduction in RT-induced side effects is reported with proton therapy [11]. Furthermore, proton therapy is thought to exhibit improved RBE due to the induction of CDD, enhancing the anti-tumor response. Comparison of intensity-modulated PT (IMPT) vs. IMRT has proved non-inferior in HNSCC patients, with a relative improvement in side effects such as dysphagia and xerostomia, but not mucositis and ORN [162,163,164]. However, early reports indicate that proton therapy may observe slightly higher rates of ORN compared to photon RT [165]. Studies evaluating both preoperative and adaptive hypofractionation RT (NCT05538533, NCT04477759) are underway, as are studies using machine learning platforms to improve RT planning and minimization of acute toxicity (NCT05979883).

### 6.2. Dietary and Nutritional Support

Due to tumor sites and treatment, HNSCC patients are at a particularly high risk of malnutrition. This can be further exacerbated by the development of nutrition impact symptoms (NIS), including oral mucositis, xerostomia, and taste alterations, which impair oral intake and contribute to significant weight loss [166]. Thus, maintaining proper nutrition in this context is important for improving treatment tolerance, reducing acute toxicities, and preserving long-term function.

While specific dietary strategies using agents such as Vitamin E, curcumin, rosmarinic acid, and other antioxidant or nutraceuticals show promise in modulating oxidative stress and fibrotic pathways [167], the prevention and management of RIF also relies on fundamental, general nutritional care [168,169]. Evidence supports the role of diet in mitigating fibrosis in organs such as the lungs and liver [170,171]. However, direct evidence for specific dietary therapies in reversing RIF is limited. Therefore, future studies evaluating nutritional strategies that ensure adequate caloric and protein intake, hydration, and symptom management could help highlight the preventative role of nutrition in maintaining tissue health and integrity [172].

Emerging evidence also suggests that interventions targeting the gut microbiota, such as nicotinamide mononucleotide (NMN) supplementation, may modulate fibrosis-related pathways and offer novel therapeutic potential [173]. However, large-scale, RIF-specific studies on dietary patterns, micronutrients, and functional foods remain insufficient. Future research should focus on defining evidence-based nutritional approaches for RIF, including the role of personalized nutrition and functional foods in supporting long-term tissue health. Special attention is needed to clarify the dosage, timing, and safety of the use of specific dietary ingredients during and after RT [167].

## 7. Conclusions and Future Perspectives

Sequencing definitive and/or adjuvant therapy, RT dose, the deleterious impact of (chemo) RT on functional parenchymal and stem cells within the microenvironment, and likely patient genetics and external environment, surely all contribute to RIF. The lack of robust models to study RIF has impaired our ability to develop better therapeutics to manage this disease and address unmet needs in the field, such as biomarker discovery, patient stratification, AI-based imaging, and preventative strategies. A number of established agents derived from retrospective studies and early Phase 2 trials have demonstrated an ability to reduce RIF and improve patient-reported outcomes. However, continued investigation into anti-fibrotic therapies demonstrating efficacy in pulmonary and skin RIF models should help to improve therapeutic response and treatment options, ultimately improving quality of life and morbidity for this cohort of oncology patients.

## Figures and Tables

**Table 1:** Acute and chronic side effects associated with RT in patients with HNSCC.

Acute	Chronic
Mucositis	Xerostomia
Dysgeusia	Trismus
Voice changes	Odynophagia
Dysphagia	Osteoradionecrosis
Lymphedema	Pharyngoesophageal stenosis
Dermatitis	Fibrosis
Parotitis	Thyroid dysfunction
	Periodontitis
	Dental caries
	Radiation-associated neoplasms
	Musculoskeletal loss of function or range of motion

## Data Availability

Data sharing is not applicable to this article.
